# Awareness and use of nonoccupational HIV post-exposure prophylaxis and factors associated with awareness among MSM in Beijing, China

**DOI:** 10.1371/journal.pone.0255108

**Published:** 2021-08-26

**Authors:** Yanming Sun, Guiying Li, Hongyan Lu

**Affiliations:** Institute for HIV/AIDS and STD Prevention and Control, Beijing Center for Disease Prevention and Control, Beijing Research Center for Preventive Medicine, Beijing, China; The Chinese University of Hong Kong, HONG KONG

## Abstract

**Background:**

Human immunodeficiency virus (HIV) sexual transmission among men who have sex with men (MSM) has increased markedly in Beijing, China, during the past decade. Nonoccupational HIV post-exposure prophylaxis (nPEP) is a highly efficacious biomedical prevention strategy that significantly reduces HIV-transmission risk. This study examined nPEP awareness among MSM and the factors influencing it.

**Methods:**

Consecutive, cross-sectional MSM surveys were conducted from April to August of 2018 and 2019. Demographic data as well as that on behavior and awareness regarding nPEP was collected. Factors influencing nPEP awareness were assessed using univariate and multivariable logistic regression.

**Results:**

There were 1,202 eligible responders recruited. Of the responders, 42.5% had nPEP awareness, and 59.9% expressed interest in receiving nPEP in the future, if required. Greater odds of nPEP awareness were associated with younger age, higher education level (adjusted odds ratio [aOR]: 4.011, 95% confidence interval [CI]: 2.834–5.678, P<0.001), higher income, use of the Internet to meet sexual partners (aOR: 2.016, 95% CI: 1.481–2.744, P<0.001), greater HIV-related knowledge (aOR: 3.817, 95% CI: 1.845–7.899, p<0.001), HIV testing (aOR: 2.584, 95% CI: 1.874–3.563, p<0.001), and sexually transmitted infections (aOR: 1.736, 95% CI: 1.174–2.569, P = 0.006). Lower odds of nPEP awareness were associated with greater stigma score (aOR: 0.804, 95% CI: 0.713–0.906, P<0.001).

**Conclusions:**

The findings indicate suboptimal awareness and low utilization of nPEP in Beijing and highlight nPEP inequities among MSM with stigma. Strengthening the training of health service providers and peer educators in reducing stigma and disseminating nPEP knowledge is imperative.

## Introduction

Sexual transmission by men that have sex with men (MSM) is the most commonly reported route of human immunodeficiency virus (HIV) transmission in Beijing and this group is disproportionately affected by HIV and accounts to 77.97% of the newly diagnosed cases [1]. During the initial years of the HIV epidemic, condom use was the only method available for preventing HIV transmission among MSM. However, a previous study demonstrated that the rate of consistent condom use during anal sex was relatively constant, without an obvious increase (approximately 60%–70%) for many years [2]. Therefore, the identification of new intervention methods to prevent the spread of HIV among MSM is of paramount importance.

The use of combined antiretroviral therapy (cART) has greatly improved the lives of people living with HIV/AIDS. More recently, considerable attention has been given to the use of cART as a strategy to prevent or control the spread of HIV transmission in several ways. It involves taking antiretroviral treatment to reduce the chances of HIV infection. In 2005, the United States Centers for Disease Control and Prevention (CDC) recommended a 28-day course of highly active antiretroviral therapy for individuals who experience nonoccupational exposure to blood, semen, or genital secretions from a known HIV-positive individual, provided they seek treatment within 72 h of exposure [3]. The cost-effectiveness of nonoccupational HIV post-exposure prophylaxis (nPEP) has been demonstrated, especially in high-risk groups [4].

Individuals who may require nPEP should be aware of its existence and be urged to request it immediately after high-risk exposure or condom failure. However, related studies in China are limited. Given that knowledge regarding nPEP is a prerequisite (although not sufficient) for acceptability, the current study sought to elucidate the behavioral, demographic, and social parameters correlating with nPEP. Identifying these parameters is essential in providing population-specific information. We aimed to study the factors associated with nPEP awareness among MSM. The results of our analysis will help policymakers conceptualize better strategies to increase the awareness and use of nPEP among MSM in Beijing.

## Materials and methods

### Study design and participants

Consecutive cross-sectional surveys were conducted in 2018 and 2019, from April to August, in Beijing. The target population included MSM aged 18 years or above who reported oral or anal sex with at least one male sex partner in the half past year. People with mental disability were excluded from the survey.

### Sample size

The sample size was calculated using simple random sampling. The sample size was 600, which ensured that the width of the 95% confidence interval (CI) would not exceed 8% for any percentage of awareness. Variance was calculated as follows:

$$V(P)=\frac{1-f}{n}\frac{1}{N-1}NP(1‐P)$$

where V is the variance, N is the population, n is the sample size, f is the sampling ratio, $\frac{N}{N-1}$ approximately equals to 1, and f = n/N approximately equals to 0.

### Sampling and recruitment

From April to August of 2018 and 2019, 13 and 11 MSM with different demographic characteristics were selected as survey seeds, respectively, and three recruitment cards were issued to each of them. They subsequently selected three other MSM from their friends to participate in the survey. The seeds had to meet the following inclusion criteria: a) be willing to recruit other MSM participants, b) have a wide range of contacts in the MSM community, and c) have a selection of seeds from across all types of demography. Seeds were selected mainly according to age, education level, and means of meeting partners, and they exhibited various types of these three aspects (Table 1). Each participant had to have a recruitment card to participate in the survey. After completing the survey, each participant received three recruitment cards. Recruitment continued until the number of participants reached approximately 600 per year.

**Table 1:** Demographic composition of the study seeds in 2018 and 2019.

Means of meeting partners^a^	2018	2019
Face-to-face directly^b^	30.8%(4/13)	45.5% (5/11)
Internet	76.9%(10/13)	90.9%(10/11)
Age		
<25	0	9.1% (1/11)
25–39	76.9%(10/13)	54.5% (6/11)
≥40	23.1%(3/13)	36.4% (4/11)
Education		
Middle school or lower	23.1%(3/13)	18.2% (2/11)
College or higher	76.9%(10/13)	81.8%(9/11)

^**a**^Some seeds identified sexual partners both online and face-to-face.

^**b**^These seeds identified sexual partners by meeting them face-to-face in bars, parks, exercise groups, etc.

After written informed consent was obtained from the participants, each subject underwent an anonymous interview to collect information. Participants were interviewed by skilled interviewers in a private room at the voluntary counseling and testing clinic of the Beijing CDC. Mobile phone numbers or email addresses were also provided by the participants for notification of laboratory testing results, when necessary. A cash reward of 50 Chinese Yuan (CNY) (approximately 7–8 United States dollars) was awarded for participation in the questionnaire survey. On recruiting three MSM, a participant would also receive a reward of 100 CNY. As a part of the national HIV sentinel surveillance program, this study was approved by the Institutional Review Board of the National Center for AIDS/STD Control and Prevention, China CDC, (IRB0000276 and FWA00002958).

### Measures

A structured questionnaire was developed by the research team. Except for nPEP-related questions, a survey questionnaire that had been previously used in annual MSM surveys in Beijing for more than 10 years was employed; nPEP-related issues were added by the research team in 2018 and pretested using 12 young adults (not included in the study).

The questionnaire was used to collect data, including demographic characteristics (age, education, marital status, residency, etc.), condom-use frequency during sexual contact, and previous HIV testing. To assess nPEP awareness, participants were asked if they had ever heard about nPEP, which was defined as medication taken orally after a sexual encounter by participants who believed that they might have been exposed to HIV. They were also asked if they had ever received nPEP. Responses were dichotomously coded (yes or no). There were eight knowledge questions in this survey, five of which were extracted from the United Nations General Assembly Special Session [5]. The statements were as follows: (1) people can protect themselves from contracting HIV by having sex with only one faithful, uninfected partner; (2) people can protect themselves from contracting HIV by using condoms; (3) a healthy-looking person can have HIV; (4) a person can acquire HIV from mosquito bites; and (5) a person can acquire HIV from sharing a meal with someone who is infected. The remaining three questions regarded the HIV transmission route: (1) a person can acquire HIV from receiving HIV-infected blood, (2) a person can acquire HIV from sharing needles with an infected individual, and (3) children born to HIV-infected women may contract HIV. Six or more correct answers were considered as “good knowledge of HIV” and less than six as “poor knowledge of HIV”. To determine the participants’ level of stigma, respondents were asked, “If a relative is part of the MSM population, will others feel ashamed?”; “Would you hide your MSM identity to avoid discrimination?”; and “Are you ashamed after having sex with other men?” Participants rated each item on a 3-point scale (0 = Never/Strongly disagree, 1 = Sometimes/partially agree, 2 = Very often/Strongly agree). The final stigma score was the sum of the three responses.

### Laboratory testing

Five-milliliter blood specimens were collected for syphilis and HIV testing from eligible MSM after the survey. The enzyme-linked immunosorbent assay (ELISA) and rapid plasma reaction methods were used to determine syphilis infection. ELISA was used for HIV screening and western blotting to confirm if the screening test was positive.

### Statistical analysis

We used SPSS Statistics (version 19.0; The EpiData Association, Odense, Denmark) to analyze the data. Factors associated with nPEP awareness were analyzed using univariable and multivariable logistic regression models by presenting odds ratio (OR) and adjusted OR (aOR), respectively. A stepwise approach was used for variable selection, and the criterion for variable entry into the multiple variable analysis was a P value less than 0.1 obtained in the univariable analysis. Statistical significance was set at P < 0.05.

## Results

### Population characteristics

Between 2018 and 2019, 1,202 MSM (602 and 600 in 2018 and 2019, respectively) were recruited. The average age of the MSM in our study was 39.4 years (SD±11.0). Table 2 demonstrates the characteristics of HIV-infection rate, demographics, condom use, HIV-testing history, and HIV knowledge among recruited MSM. Of these, 42.0% had a college degree or higher. Homosexual orientation was observed in 65.1% of the participants. Consistent condom use in sexual intercourse during the preceding 6 months was observed in 60.7% of our study population, and the self-reported history of contracting sexually transmitted infections (STIs) was 16.1%. Of the surveyed MSM, 66.4% were tested for HIV within the preceding 12 months. The HIV-infection rate was 7.8%. During the study, 79.8% of the MSM received peer-educator support. HIV knowledge was identified in 90.6% of the MSM in our study. The average stigma score was 3.7 (SD±1.2).

**Table 2:** Demographic and behavioral characteristics of MSM recruited in 2018 and 2019 in Beijing, China (N = 1202).

	n	%
Age (years)		
<25	70	5.8
25–39	583	48.5
≥40	549	45.7
Marital status		
Single	621	51.7
Married/cohabiting	434	36.1
Divorced/widowed	147	12.2
Education		
Middle school or lower	697	58.0
College or higher	505	42.0
Time in Beijing (years)		
≥2	1028	85.5
<2	174	14.5
Monthly income (CNY)		
<5,000	622	51.8
5,000–10,000	319	26.5
≥10,000	261	21.7
Sexual orientation		
Bisexual	420	34.9
Homosexual	782	65.1
Main means of meeting partners		
Face-to-face directly	474	39.4
Internet	728	60.6
Self-assessed risk of HIV infection		
Low	923	76.8
High	279	23.2
Awareness of nPEP	511	42.5
Previous nPEP use	21	1.7
No of MSM partners in the preceding 6 months		
1	358	29.8
2–9	707	58.8
≥10	137	11.4
Condom use in MSM anal sex during the preceding 6 months ^a^		
Consistent	615	60.7
Inconsistent	398	39.3
Rush poppers used during the preceding 6 months	357	29.7
STI history	194	16.1
Peer education in preceding year	959	79.8
HIV testing in the preceding 12 months	798	66.4
HIV positive	94	7.8
Syphilis positive	72	6.0
Good knowledge of HIV	1089	90.6
	Mean	SD
Stigma^b^	3.7	1.2

^a^1,013 MSM had MSM anal sex during the preceding 6 months

^b^measured on a 7-point scale from 0 to 6, 6 = extremely stigma.

### Awareness, knowledge, and use of nPEP

Of the study participants, 42.5% reported having heard of nPEP. A total of 21 respondents (1.7%) reported previous nPEP use (Table 2). Table 3 shows the knowledge of nPEP among MSM. A total of 59.9% indicated that they would use nPEP in the future, if required. Of the MSM, 67.3% knew where to purchase nPEP medication. However, more than 60% of the MSM were not aware of the timing and duration of nPEP intake. The main reason for using nPEP was their involvement in high-risk sexual intercourse (85.7%).

**Table 3:** Knowledge of nPEP among MSM recruited in 2018 and 2019 in Beijing, China (n = 511)^a^.

	N	%
Where to purchase nPEP		
Known	344	67.3
Unknown	167	32.7
Timing of nPEP initiation after unprotected sex		
<72 h	203	39.7
Unknown	308	60.3
Medication duration		
28 days	181	35.4
Unknown	330	64.6
Likelihood of using nPEP		
Yes	306	59.9
No/unsure	205	40.1
Reason why nPEP was sought^b^		
Related to sexual practices	18	85.7
Not related to sexual practices	3	14.3

^a^Calculated for those who knew about nPEP.

^b^Calculated for those who had used nPEP.

### Factors related to nPEP awareness

Univariate and multivariate analyses were performed to explore the possible factors associated with nPEP awareness (Table 4). In the univariate analysis, we found the following factors to be associated with increased nPEP awareness: (1) age under 40 years (OR for 25–39 years of age was 3.493, 95% CI: 2.721–4.485, P<0.001, OR and that for age under 25 years 3.066, 95% CI: 1.849–5.085, P<0.001), (2) living in Beijing for over two years (OR: 1.893, 95% CI: 1.338–2.678, P<0.001), (3) attended college-level education and/or higher (OR: 9.483, 95% CI: 7.270–12.368, P<0.001), (4) higher monthly income (OR for income of 5,000–10,000 CNY was 2.982, 95% CI: 2.242–3.966, P<0.001, and OR that of 10,000 CNY and above 9.754, 95% CI: 6.955–13.680, P<0.001), (5) homosexual orientation versus bisexual (OR: 2.300, 95% CI: 1.788–2.959, P<0.001), (6) use of the Internet as the main way of meeting sexual partners (OR: 3.366, 95% CI: 2.615–4.334, P<0.001), (7) having good HIV knowledge (OR:8.776, 95%CI: 4.536–16.980, P<0.001), (8) use of rush poppers during the preceding 6 months (OR: 2.670, 95% CI: 2.071–3.442, P<0.001), (9) HIV-testing in the preceding 12 months (OR: 2.607, 95% CI: 2.014–3.375, P<0.001), (10) infection with an STI (OR: 1.793, 95% CI: 1.316–2.444, P<0.001), and (11) participation in peer-education programs on HIV prevention in the preceding 12 months (OR: 2.505, 95% CI: 1.830–3.428, P<0.001). MSM who were married/cohabitating and divorced/widowed were less likely to be aware of nPEP (OR: 0.264, 95% CI: 0.202–0.344, P<0.001, and OR: 0.310, 95% CI: 0.210–0.457, P<0.001, respectively). Those with HIV and syphilis infection (OR: 0.520, 95% CI: 0.327–0.825, P = 0.006, and OR: 0.538, 95% CI: 0.319–0.906, P = 0.020, respectively) and higher stigma score (OR: 0.637, 95% CI: 0.574–0.708, P<0.001) were also less likely to acquire nPEP knowledge.

**Table 4:** Factors associated with the nPEP awareness among MSM recruited in 2018 and 2019 in Beijing, China.

	Awareness		Unawareness		Univariate Analysis		Multivariate Analysis	
Categorical variables	N	%	N	%	OR (95%CI)	P	aOR(95%CI)	P
Age (years)								
≥40	147	26.8	402	73.2	1		1	
25–39	327	56.1	256	43.9	3.493(2.721–4.485)	<0.001	1.711(1.246–2.351)	0.001
<25	37	52.9	33	47.1	3.066(1.849–5.085)	<0.001	2.343(1.274–4.308)	0.006
Marital status								
Single	355	57.2	266	42.8	1			
Married/cohabitating	113	26.0	321	74.0	0.264(0.202–0.344)	<0.001	NS^a^	
Divorced/widowed	43	29.3	104	70.7	0.310(0.210–0.457)	<0.001	NS	
Time in Beijing (years)								
<2	52	29.9	122	70.1	1			
≥2	459	44.6	569	55.4	1.893(1.338–2.678)	<0.001	NS	
Education								
Middle school or lower	148	21.2	549	78.8	1		1	
College or higher	363	71.9	142	28.1	9.483(7.270–12.368)	<0.001	4.011(2.834–5.678)	<0.001
Monthly Income (CNY)								
<5,000	154	24.8	468	75.2	1		1	
5,000–10,000	158	49.5	161	50.5	2.982(2.242–3.966)	<0.001	1.471(1.033–2.095)	0.032
≥10,000	199	76.2	62	23.8	9.754(6.955–13.680)	<0.001	2.801(1.809–4.337)	<0.001
Sexual orientation								
Bisexual	125	29.8	295	70.2	1			
Homosexual	386	49.4	396	50.6	2.300(1.788–2.959)	<0.001	NS	
Main means of meeting partners								
Face-to-face directly	121	25.5	353	74.5	1		1	
Internet	390	53.6	338	46.4	3.366(2.615–4.334)	<0.001	2.016(1.481–2.744)	<0.001
Self-assessed risk of HIV infection								
Low	390	42.3	533	57.7	1			
High	121	43.4	158	56.6	1.047(0.799–1.372)	0.741	/	
Knowledge of HIV								
Poor	10	8.8	103	91.2	1		1	
Good	501	46.0	588	54.0	8.776(4.536–16.980)	<0.001	3.817(1.845–7.899)	<0.001
No of MSM partners in the preceding 6 months								
1	159	44.4	199	55.6	1			
2–9	302	42.7	405	57.3	0.933(0.722–1.206)	0.597	/	
≥10	50	36.5	87	63.5	0.719(0.480–1.079)	0.111	/	
Condom use in MSM anal sex during the preceding 6 months^b^								
Consistent	291	47.3	324	52.7	1			
Inconsistent	173	43.5	225	56.5	0.856(0.664–1.103)	0.230	/	
Rush poppers used during the preceding 6 months								
No	299	35.4	546	64.6	1			
Yes	212	59.4	145	40.6	2.670(2.071–3.442)	<0.001	NS	
HIV testing in the preceding 12 months								
No	112	27.7	292	72.3	1		1	
Yes	399	50.0	399	50.0	2.607(2.014–3.375)	<0.001	2.584(1.874–3.563)	<0.001
STI history								
No	405	40.2	603	59.8	1		1	
Yes	106	54.6	88	45.4	1.793(1.316–2.444)	<0.001	1.736(1.174–2.569)	0.006
Peer education in preceding 12 months								
No	63	25.9	180	74.1	1			
Yes	448	46.7	511	53.3	2.505(1.830–3.428)	<0.001	NS	
HIV								
N**e**gative	484	43.7	624	56.3	1			
positive	27	28.7	67	71.3	0.520(0.327–0.825)	0.006	NS	
Syphilis								
N**e**gative	490	43.4	640	56.6	1			
positive	21	29.2	51	70.8	0.538(0.319–0.906)	0.020	NS	
Continuous variable	Mean	SD	Mean	SD				
Stigma score^c^	3.3	1.4	4.0	1.0	0.637(0.574–0.708)	<0.001	0.804(0.713–0.906)	<0.001

^a^ NS: non-significant.

^b^ 1013 MSM has MSM anal sex during the past six months.

^c^ Measured on a 7-point scale from 0 to 6, 6 = extremely stigma.

Multivariate logistic regression showed that MSM were two fold more likely to be aware of nPEP if they were younger (aOR for 25–39 years of age was 1.711, 95% CI: 1.246–2.351, p = 0.001, and that for age under 25 years was 2.343, 95% CI: 1.274–4.308, p = 0.006). MSM with high income were more likely to hear about nPEP (aOR: 1.471, 95% CI: 1.033–2.095, p = 0.032, and aOR: 2.801, 95% CI: 1.809–4.337, p<0.001). In addition, other factors, including having a college or higher degree (aOR: 4.011, 95% CI: 2.834–5.678, P<0.001), having used the Internet as their main way of meeting sexual partners (aOR: 2.016, 95% CI: 1.481–2.744, P<0.001), having good HIV knowledge (aOR: 3.817, 95% CI: 1.845–7.899, p<0.001), HIV testing in the preceding 12 months (aOR: 2.584, 95% CI: 1.874–3.563, p<0.001), and infection with STIs (aOR: 1.736, 95% CI: 1.174–2.569, P = 0.006) were found to be associated with increased nPEP awareness. MSM who had greater stigma score (aOR: 0.804, 95% CI: 0.713–0.906, P<0.001) were less likely to hear about nPEP.

## Discussion

In the present study, 42.5% of MSM had heard about nPEP, and 1.7% had previously received nPEP. Knowledge of nPEP varies widely by geographical region; the rate of nPEP awareness among the present MSM was higher than that reported in Spain between 2009 and 2010 (34%) [6], San Juan between 2010 and 2012 (16%) [7], and New York from 2006 to 2007 (36%) [8]; however, it was similar to that reported in Pittsburgh between 2010 and 2012 (47%) [7] and lower than that reported in Boston between 2010 and 2012 (64%) [7], Italy in 2014 (82.3%) [9], New York in 2017 (80.2%) [10], and Africa in 2015 (71.1%) [11]. Although the awareness rate in some areas was lower than ours, most of these studies had small sample sizes or earlier survey times, rendering it difficult for their rates to be consistent with the current ones.

The prevalence of nPEP use was similar to that in Spain between 2009 and 2010 (2.6%) [6], Finland in 2010 (1.3%) [12], and the United States in 1999 (1.9%) [13]; however, it was lower than that in the United States (4.4%) in 2013 [14] and Canada between 2009 and 2012 (8.8–28.7%) [15]. In Beijing, the lack of an nPEP-promotion campaign, which had been undertaken only in recent years and is not widely available as nPEP is prescribed legally in only one Infectious Disease Hospital in Beijing. There has been no structured effort to broadcast its existence to the population. Previous studies have shown that these campaigns can promote the awareness and use of PEP in the future [6–8, 13].

In the analysis of the factors associated with nPEP awareness, we found that MSM with younger age, in general; a high level of education; and high income were more likely to be aware of nPEP probably due to superior knowledge regarding biomedical prevention. However, in similar studies in other countries, socioeconomic status was not a factor for nPEP awareness [10, 11]. This may be related to the unique national conditions and policies regarding the promotion of nPEP or a strong understanding and acceptance of new modalities; therefore, further research is warranted. Conditions associated with living in poverty, such as crowding, underemployment, financial and other stress, and exposure to violence, may increase HIV-risk behavior [16–20]. Therefore, targeting MSM of lower socioeconomic status understand and use nPEP is an issue that should be urgently addressed. A closer association with the MSM culture, such as having low levels of stigma related to sexual orientation, is also related to greater nPEP awareness [6]. Stigma can be perceived as a threat to social identity among MSM. The negative impact of stigma on nPEP awareness may be explained by social isolation and a lack of supportive networks. This corroborates the findings of other studies [9, 10, 21, 22]. Individuals living with high stigma were also less likely to have received nPEP or have had access to prescribers and providers of nPEP [21].

MSM constitute a hidden population, and it is difficult to disseminate health-related information through mass media. LGBTQ-related or institutional websites have become the main channels of awareness. Since 2018, the China CDC has implemented programs and mobilized funding to support the Beijing Health Department and nongovernmental organizations in nPEP implementation. They have also provided extensive nPEP information online, which can be an effective resource for education/outreach programs. It is possible that MSM who use the Internet as their main way of meeting sexual partners may also frequent LGBTQ-related or institutional websites where information regarding preventive measures exists. The use of the Internet for meeting partners was certainly associated with greater nPEP awareness in the present study, and this finding has also been reported by other authors [6, 8].

Survey participants who had undergone HIV-testing in the previous year had, therefore, received a certain degree of HIV-related counseling, which ideally should include education regarding prevention. nPEP awareness among MSM who have been tested is 2.584 times that among those untested. Consistent with previous studies, previous HIV testing could be a marker for higher awareness for HIV risky behavior or reflect previous counselling [8]. Similarly, previous STD infection can also increase the awareness of nPEP, which indicates nPEP knowledge received during healthcare consultation. However, approximately 50% of the MSM who had been tested for STIs or HIV infection remained unaware of nPEP, highlighting a missed opportunity for targeted counseling.

Previous studies have indicated that the links between sexual behavior and nPEP awareness are contradictory and inconclusive [6, 8]. In our study, compared with self-assessed individuals at lower risk of HIV infection, MSM who are at high risk do not have better knowledge regarding nPEP. Further, nPEP awareness did not increase with the number of partners and/or non-association with condom or aphrodisiac use during sexual behavior. Therefore, high-risk groups in need of greater nPEP protection do not have the relevant requisite knowledge.

Therefore, nPEP awareness remains suboptimal and is probably not reaching MSM most likely to benefit from nPEP. To resolve this issue, the following should be considered: First, in the current era of universal HIV testing, providers should also integrate the sexual history of all patients, as understanding patients’ sexual practices can facilitate the appropriate targeting and delivery of HIV prevention and counseling. Second, in our study, peer education did not play an effective role in the dissemination of nPEP knowledge. As shown in other studies, well-trained peer educators are critical in terms of delivering accurate knowledge on HIV [23] and promoting harm-reduction interventions (e.g., condom use, encouraging regular HIV testing, and psychological support) through outreach activities [23, 24]. Therefore, as nPEP is a relatively novel HIV prevention method, health departments should target counseling providers and peer educators for training on nPEP education and referral resources. This may help improve awareness of and access to nPEP for at-risk MSM.

One encouraging finding is that nPEP seems to be very appealing to MSM. Sixty percent of MSM who knew about nPEP indicated that they would likely use nPEP in the future, demonstrating consistency between knowledge, willingness, and actions. It also suggests that lack of awareness is a primary barrier. Unfortunately, more than 30% of MSM do not know where to purchase nPEP medicine, and more than 60% do not know the correct timing for initiating and concluding nPEP therapy. Low rates of nPEP utilization suggest that, even if MSM are aware of nPEP, they may still find it difficult to either recognize the high risk underlying their behavior or identify sources of nPEP medication, since nPEP entails self-identification of a high-risk exposure and healthcare-seeking behavior within 72 h for optimal chemoprotection. This indicates that basic medication information should be included in the nPEP promotion program [14].

Our study had certain limitations. First, due to the cross-sectional design, we could only evaluate the association and not the causality of the risk factors for nPEP awareness. Second, it was difficult to implement randomization. Generally, we applied a convenience sampling method; hence, our results might not have been representative of the entire MSM population in Beijing. Third, in our study, behavioral information relied on self-reporting, which might have been influenced by recall and social-desirability bias.

In conclusion, we found suboptimal awareness and low utilization of nPEP in Beijing. Most MSM were willing to use nPEP in the future, if necessary. It is critical to strengthen the training of healthcare providers and peer educators to improve the dissemination of nPEP knowledge.

## Supporting information

S1 File (DOCX)

S2 File (XLSX)
