# Supplementary material for: Awareness and use of nonoccupational HIV post-exposure prophylaxis and factors associated with awareness among MSM in Beijing, China
Source: PLoS One. 2021 Aug 26;16(8):e0255108. doi: 10.1371/journal.pone.0255108 (PMC8389520; doi:10.1371/journal.pone.0255108)
Supplement: S1 File — (DOCX) [file pone.0255108.s001.docx]

调查问卷

1. 问卷编号______
2. 调查日期_____年_____月_____日
3. 出生日期_____年_____月_____日
4. 婚姻状况 ①未婚 ②在婚 ③同居 ④丧偶/离异
5. 本市居住时间 ①＜3个月 ②3-6个月 ③ 7-12个月 ④1-2年 ⑤2年以上
6. 文化程度 ①文盲 ②小学 ③初中 ④高中或中专 ⑤大专及以上
7. 月收入 ①＜1000 ②1000-2000 ③2000-3000 ④3000-4000 ⑤4000-5000 ⑥5000-10000 ⑦≥10000 ⑧无收入
8. 性取向 ①男性 ②女性 ③双性 ④其它__________
9. 寻找性伴最常的方式 ①酒吧/歌舞厅 ②桑那/浴池/按摩 ③公园/公厕

④互联网 ⑤运动组 ⑥朋友介绍 ⑦茶室/会所 ⑧其它__________

1. 认为自己感染危险多大？ ①没有 ②低 ③有点高 ④很高

-------------------------------------------------------------------------------------

艾滋病相关知识

1. 一个感染了艾滋病病毒的人能从外表上看出来吗？ ①能 ②不能 ③不知道
2. 输入带有艾滋病病毒的血液/血制品会得艾滋病吗？ ①会 ②不会 ③不知道
3. 与艾滋病病毒感染者共用针具可能得艾滋病吗？ ①可能 ②不可能 ③不知道
4. 正确使用安全套可以减少艾滋病的传播吗？ ①可以 ②不可以 ③不知道
5. 只与一个性伴发生性行为可以减少艾滋病的传播吗？ ①可以 ②不可以 ③不知道
6. 感染艾滋病病毒的妇女生下的小孩有可能得艾滋病吗？ ①可能 ②不可能 ③不知道
7. 与艾滋病病毒感染者/病人一起吃饭会感染艾滋病吗？ ①会 ②不会 ③不知道
8. 蚊虫叮咬会传播艾滋病吗？ ①会 ②不会 ③不知道

-------------------------------------------------------------------------------------

nPEP相关知识

1. 听说过艾滋病阻断药么？ ①知道 ②不知道（跳至29）
2. 你吃过么？ ①吃过 ②没吃过（跳至23）
3. 为什么去吃？①对方HIV阳性，无套行为 ②怀疑对方阳性，无套行为

③其他原因______________

1. 什么时间吃的？ _______年____月___日
2. 你知道从哪里可以拿到阻断药？ ①艾滋病定点治疗医院 ②药店 ③网上

④其他，注明____________________ ⑤不知道

1. 需要连续吃多久？ ①15天 ②28天 ③28天以上 ④不知道
2. 你觉得大概需要花多少钱？ ①3000以下 ②3000-5000 ③5000-7000 ④7000以上 ⑤不知道
3. 如果和陌生人无套行为后，你会去吃阻断药么？ ①会吃（跳至28） ②可能会可能不会，说不好 ③不会吃
4. 不吃原因 ①贵 ②侥幸心理 ③其他___________________
5. 不安全行为后，最迟多久开始服药？ ①2小时 ②12小时 ③24小时 ④72小时 ⑤不知道

-------------------------------------------------------------------------------------

1. 家中有MSM，其他人会觉得难堪么？ ①不会 ②有点儿 ③肯定会
2. 为避免歧视，你会掩饰自己的MSM身份么？ ①不会 ②有点儿 ③肯定会
3. 半年内同性性行为后，你是否有感到羞耻？ ①不会 ②有点儿 ③肯定会
4. 最近六个月您的男性性伴数______
5. 最近六个月，你与同性发生过肛交吗？ ①是 ②否（跳至36）
6. 最近一次肛交时使用安全套了吗？ ①是 ②否
7. 最近六个月发生肛交时安全套使用频率？ ①从未 ②有时 ③每次
8. 最近六个月是否使用过助性剂Rush？ ①是 ②否
9. 曾经患过性病么？ ①是，性病种类__________ ②否
10. 最近一年是否接受过同伴教育？ ①是 ②否
11. 最近一年是否接受过HIV检测？①是 ②否（跳至41）
12. 检测结果 ①阴性 ②阳性 ③不确定
13. 是否吸毒？ ①是，种类__________ ②否（结束问卷）
14. 是否共用针具？①是 ②否

Questionnaire

1. ID______
2. Survey date______(yyyy/mm/dd)
3. Birthday______(yyyy/mm/dd)
4. Marital status ①Single ②Married ③Cohabitation ④Widowed/divorced
5. Time in Beijing ①<3 months ②3-6 months ③ 7-12 months ④1-2years ⑤≥2years
6. Education ①Illiteracy ②Primary school ③Junior high school ④High school / technical secondary school ⑤College degree and above
7. Monthly income (CNY) ①＜1000 ②1000-2000 ③2000-3000 ④3000-4000 ⑤4000-5000 ⑥5000-10000 ⑦≥10000 ⑧No income
8. Sexual orientation ①Homosexual ②Heterosexual ③Bisexual ④Other_________
9. Main means of meeting partners ①Bar/ Karaoke ②Sauna/bath/massage ③Park/Public Toilet ④Internet ⑤Exercise group ⑥Friend introduced ⑦Tea room/club ⑧Other__________
10. Self-assessed risk for HIV infection ①No ②Low ③A little high ④High

---------------------------------------------------------------------------------------------------------------------

Knowledge of HIV

1. A healthy-looking person can have HIV. ①Yes ②No ③Unknow
2. A person can get HIV from entering blood with HIV. ①Yes ②No ③Unknow
3. A person can get HIV from sharing syringes with someone who is infected. ①Yes ②No ③Unknow
4. People can protect themselves from contracting HIV by using condoms. ①Yes ②No ③Unknow
5. People can protect themselves from contracting HIV by having sex with only one faithful uninfected partner. ①Yes ②No ③Unknow
6. Children born to HIV-infected women may get HIV. ①Yes ②No ③Unknow
7. A person can get HIV from sharing a meal with someone who is infected. ①Yes ②No ③Unknow
8. A person can get HIV from mosquito bites. ①Yes ②No ③Unknow

---------------------------------------------------------------------------------------------------------------------

Knowledge of nPEP

1. Have you heard of non-occupational post-exposure prophylaxis (nPEP)?

①Yes ②No (Jump to 29)

1. Have you ever taken? ①Yes ②No (Jump to 23)
2. Why have you taken? ①No condom sex with HIV-positive people ②No condom sex with people with unknown HIV status ③Other reasons,______________
3. When did you take？______ （yyyy/mm/dd）
4. Where to get nPEP ? ①Hospital ②Pharmacy ③Internet

④Other,______________ ⑤Unknow

1. The duration of nPEP ①15 days ②28 days ③>28 days ④Unknow
2. How much does nPEP cost (CNY) ? ①<3000 ②3000-5000 ③5000-7000

④>7000以上 ⑤Unknow

1. Would you take nPEP if you have sex without condom ? ①Yes (Jump to 28) ②May or may not, not good to say ③No
2. Reasons for not taken ①expensive ②Lucky psychology, will not be infected

③Other reasons,______________

1. Timing of nPEP initiation after unprotected sex ①2 hours ②12 hours ③24 hours

④72 hours ⑤Unknow

---------------------------------------------------------------------------------------------------------------------

1. If a relative is part of the MSM population, will others feel ashamed?

①Never/Strongly disagree ② Sometimes/partially agree ③Very often/Strongly agree

1. Would you hide your MSM identity to avoid discrimination?

①Never/Strongly disagree ② Sometimes/partially agree ③Very often/Strongly agree

1. Are you ashamed after having sex with other men?

①Never/Strongly disagree ② Sometimes/partially agree ③Very often/Strongly agree

---------------------------------------------------------------------------------------------------------------------

1. The number of male partners during the preceding 6 months______
2. Anal sex with men during the preceding 6 months? ①Yes ②No (Jump to 36)
3. Condom use in the last anal sex ①Yes ②No
4. Condom use in anal sex during the preceding 6 months ①never ②sometimes ③every time
5. Have you use Rush poppers during the preceding 6 months? ①Yes ②No
6. Have you ever infect STDs？ ①Yes，STDs name____________ ②No
7. Have you receive peer education in preceding 12 months？ ①Yes ②No
8. Have you test for HIV in preceding 12 months？①Yes ②No (Jump to 41)
9. Test results ① negative ②positive ③uncertain
10. Have you ever taken drugs? ①Yes, drugs name____________ ②No (End)
11. Have you shared syringes? ①Yes ②No
